# Supplementary material for: Validating aggregative soil sampling using bootie and drag swabs hydrated with simple wetting agents in commercial produce fields
Source: Microbiol Spectr. 2026 Feb 2;14(3):e01663-25. doi: 10.1128/spectrum.01663-25 (PMC12955488; doi:10.1128/spectrum.01663-25)
Supplement: Supplemental figures and tables — Figures S1 to S5 and Tables S1 and S2. [file spectrum.01663-25-s0001.docx]

# Supplemental Tables

**Supplemental Table 1:** False Discovery Rates on the differences between the overall Bootie-Grab Log_10_ (CFU/g) or Drag-Grab Log_10_ (CFU/g) for indicator organisms by commodity (*p* = 0.05).

|  | **Aerobic Plate Count (APC)** **± SD, Log_10_ (CFU/g)** | | **Total Coliforms (TC) ± SD, Log_10_ (CFU/g)** | |
| --- | --- | --- | --- | --- |
| Soil from Given Commodity | Bootie – Grab | Drag - Grab | Bootie - Grab | Drag - Grab |
| Melons | _0.00013_ | ^0.00013^ | ^0.00013^ | ^0.00013^ |
| Beets | ^0.00025^ | ^0.018421^ | ^0.00013^ | ^0.0001^ |
| Leafy Greens | ^0.00013^ | ^0.00059^ | ^0.00013^ | ^0.00013^ |
| Peppers | ^0.24290^ | ^0.00013^ | ^0.00013^ | ^0.00067^ |
| Apples | ^0.00013^ | ^0.00013^ | ^0.00013^ | ^0.00013^ |

**Supplemental Table 2:** Indicator organism results by agricultural commodity soil type and collection method.

|  | **Aerobic Plate Count (APC)** **± SD, Log_10_ (CFU/g)** | | | **Total Coliforms (TC) ± SD, Log_10_ (CFU/g)** | | |
| --- | --- | --- | --- | --- | --- | --- |
| Soil from Given Commodity | Bootie | Drag | Grab | Bootie | Drag | Grab |
| Melons | 8.15±0.11_A_ | 8.04±0.11_A_ | 6.41±0.16_A_ | 7.27±0.12_A_ | 7.54±0.12_A_ | 2.21±0.17_B_ |
| Beets | 7.13±0.10_B_ | 6.93±0.10_B_ | 6.51±0.14_A_ | 6.09±0.14_C_ | 5.62±0.14_C_ | 3.59±0.20_A_ |
| Leafy Greens | 7.36±0.12_B_ | 7.21±0.12_B_ | 6.53±0.17_A_ | 6.60±0.16_B_ | 6.21±0.16_B_ | 3.97±0.22_A_ |
| Peppers | 6.52±0.07_C_ | 6.97±0.07_B_ | 6.38±0.10_A_ | 5.38±0.23_D_ | 4.94±0.23_D_ | 3.61±0.33_A_ |
| Apples | 8.07±0.07_A_ | 7.40±0.07_B_ | 6.24±0.10_A_ | 6.94±0.1_A_ | 6.26±0.13_B_ | 3.64±0.19_A_ |

_A, B, C, D_ ANOVA to compare the mean APC or TC by commodity soil type (melons, beets, leafy greens, peppers, apples) for each collection method (booties, drags, grabs). Means within the same column not sharing the same subscript letter indicate values are significantly different (p<0.05) by Post-hoc Tukey’s HSD test.

# Supplemental Figures

**Supplemental Figure 1:** Family relative abundance for sample collection method and wetting agent of the 10 taxa detected with greatest relative abundance. “Other” is defined as any identified families detected but were not in the 10 defined taxa, or any unclassified taxa in the samples.


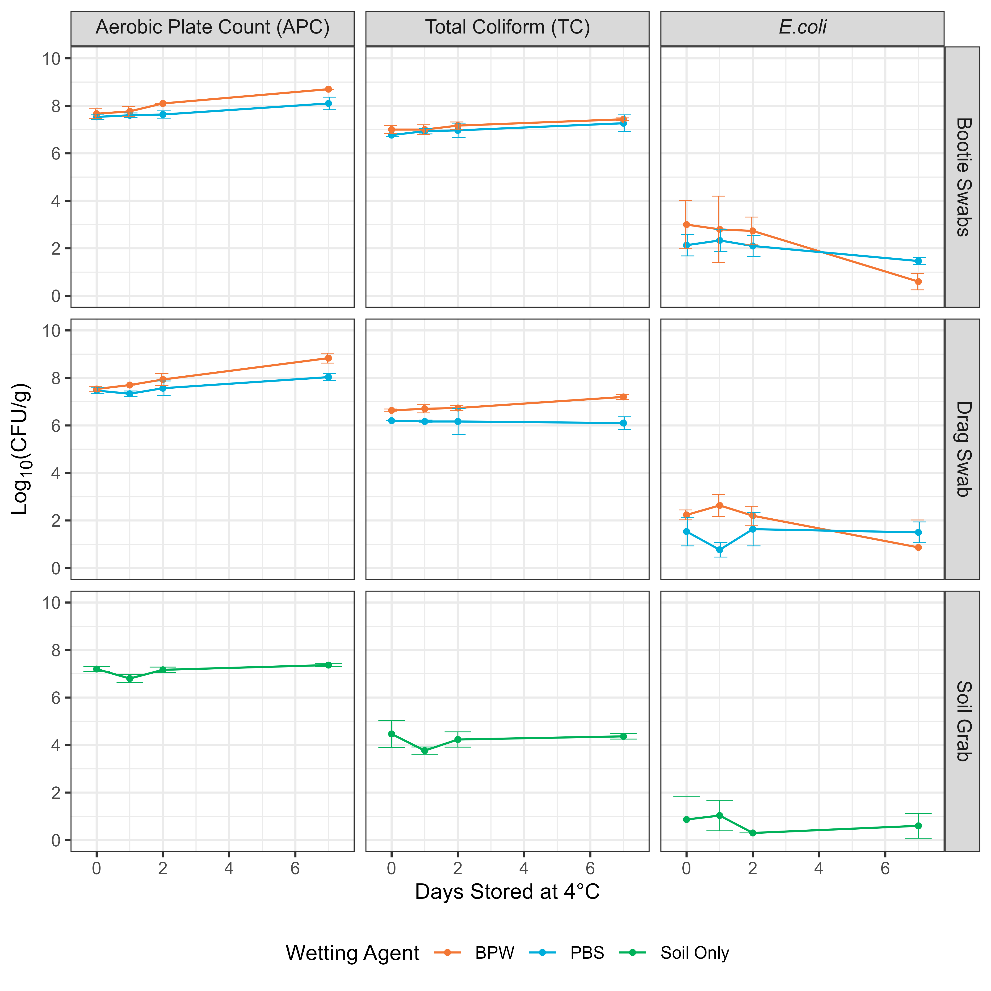


**Supplemental Figure 2:** A brief experiment was conducted to evaluate the stability of the samples in refrigerated storage (4°C) over the duration of a week. Samplers collected n=12 repetitions from the mixed horticultural field over the same walking path (Zone 1) and removed 3 randomly assigned repetitions on each of the following days; the day of sampling (day 0), day 1, day 2, and day 7 from storage at 4°C, and processed according to sample enumeration protocols. After counting the plates, an analysis of variance (ANOVA) was conducted, and a Tukey’s HSD was done to understand if there was a significant difference in change over time for APC, TC, and *E. coli* for each sample type and wetting agent. There was no significant difference (*p* > 0.05) in APCs, TC, and generic *E. coli* Log_10_ (CFU/g) between days 0, 1, and 2 for all sample collection methods and wetting agents. There was no biologically meaningful difference (<1 Log_10_ (CFU/g)) in APCs, TC, and *E. coli* Log_10_ (CFU/g) between days 0, 1, and 2 for all sample collection methods and wetting agents. There was a significant change (*p* < 0.05) between day 2 and day 7 of APC (increased) for drags and booties, both wetting agents, *E. coli* (decreased) for drags hydrated in BPW, and TC (increased) for booties and drags hydrated with BPW. This means that for most reliable results, samples must be processed within 48 hours of collection, and the samples collected from the melon farm that were processed after 36 hours of storage were acceptable for further analysis.

**Supplemental Figure**
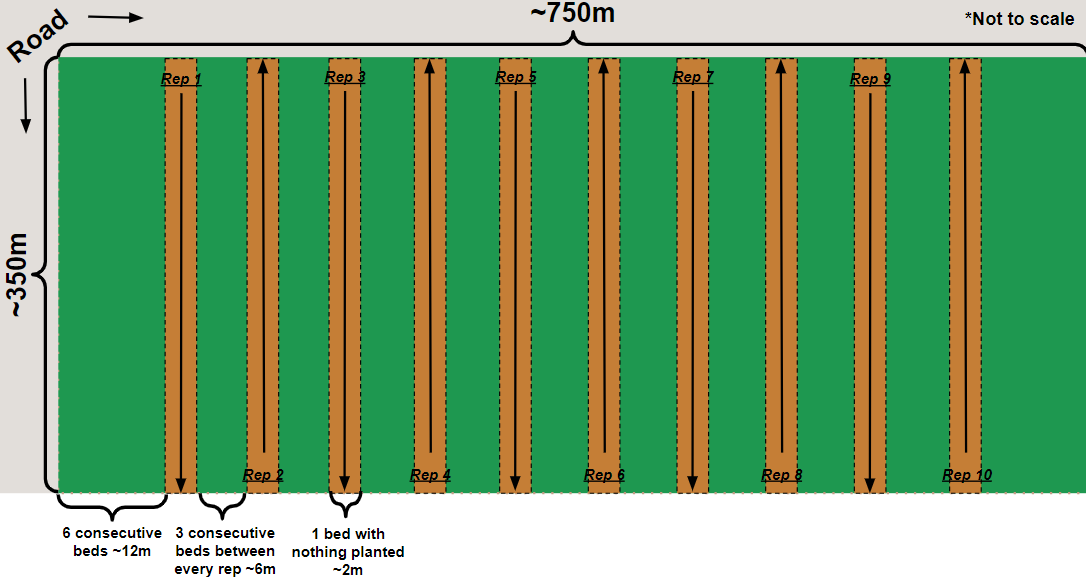
 **3: Dimensions and walking path of melon field.** A commercial melon field in the continental United States. The designated sampling area was ~750m wide by ~350m long. Each sampler walked down 1 bed (~2m wide) in one direction, and collected the following as 1 repetition; 1 boot swab hydrated in PBS, 1 boot swab hydrated in BPW, 1 drag swab hydrated in PBS, 1 drag swab hydrated in BPW, and 1 aggregate soil grab, that consisted of the sampler stopping 6 times in one repetition to take a small scoop of soil, approximately 50g each time. The sampler then reset and moved onto the next empty bed and collected the next repetition on the way back. This process was completed until 10 repetitions were completed and was repeated 24 hours later following the same paths, equaling a total on n=20 repetitions on the melon field.

**
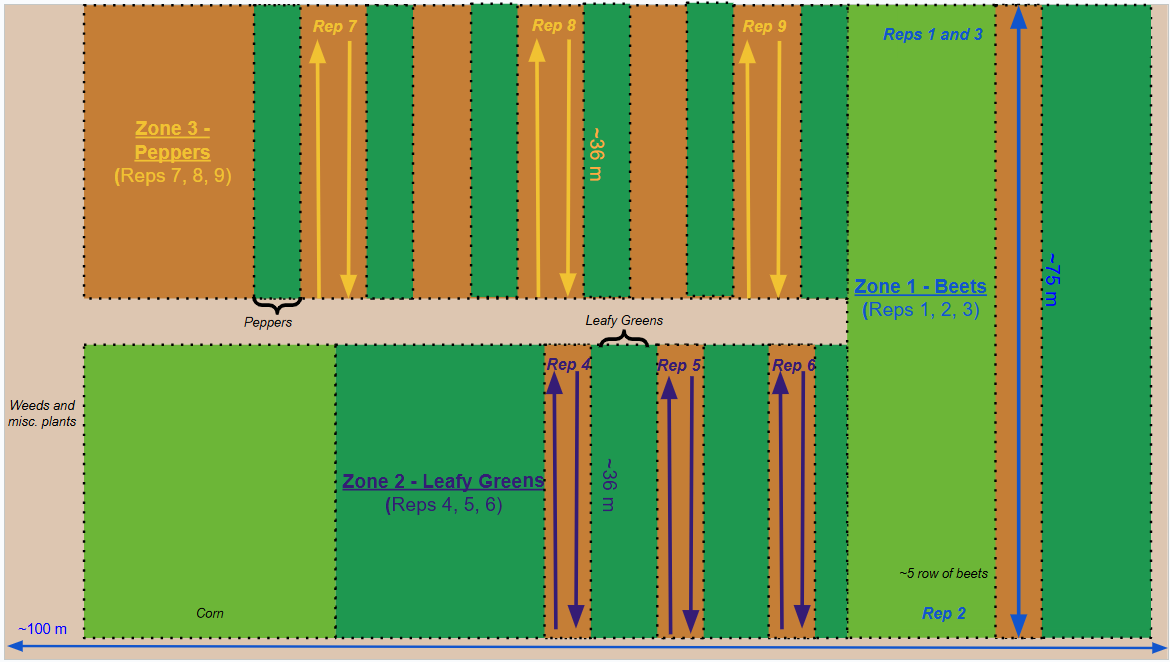
**

**Supplemental Figure 4: Dimensions and paths of beets, peppers, and leafy greens.** Beets, peppers, and leafy greens are all grown on the same small, sustainable farm, that is approximately 100 m wide and 75 m long. As the commodities were grown and irrigated in sections, samplers were able to treat each commodity as a different “zone.” Zone 1 consisted of 3 repetitions walking down the same path, approximately 75 m long, where each change in direction was 1 repetition, and was done n=3 times. Zone 2 was the location of the leafy greens. Due to the planting pattern of this farm, samplers had to divide Zone 2 into 3 walking paths, where walking down and back on the same path was 1 repetition a total of n=3 times, spacing it equally between 3 rows of leafy greens, totaling ap-proximately 72 m for each repetition. Zone 3 was conducted in a similar manner to Zone 2 for the pepper plants. This sampling sequence was repeated 14 days later, totaling n=6 repetitions for each commodity (beets, leafy greens, or peppers).

**
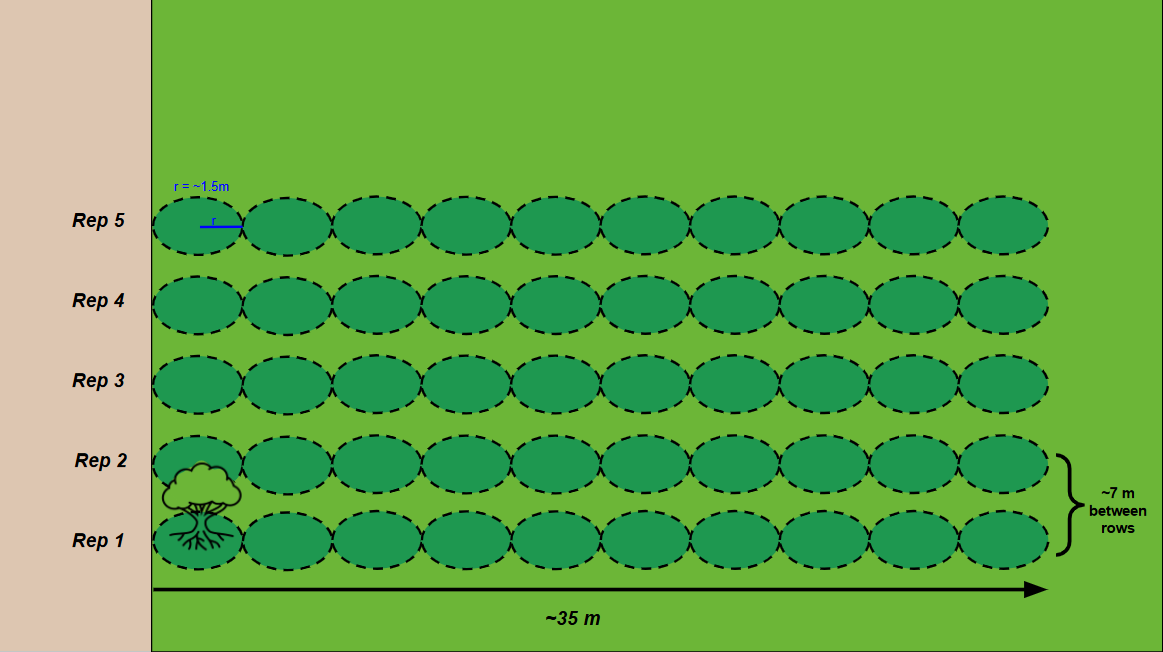
**

**Supplemental Figure 5: Dimension and walking paths of southern IL orchard.** The samplers sampled 5 consecutive rows of apple trees in an orchard. Each row of trees was approximately 7 m apart, and the length of 10 consecutive trees was approximately 35m, where trunk to trunk each tree was approximately 3 m apart.
